# Genome-Wide Identification and Expression Analysis of the *ARF* Gene Family in Chickpea (*Cicer arietinum*)

**DOI:** 10.3390/plants15111708

**Published:** 2026-05-31

**Authors:** Hanyan Feng, Yuqi Fang, Xiangtao Yang, Yirong Zhu, Zhirui Hu, Qiyi Chen, Lan Mu, Juan Li, Jianghua Chen, Dan Zong, Liangliang He

**Affiliations:** 1 College of Biological Science and Food Engineering, Southwest Forestry University, Kunming 650224, China; fenghanyan0923@swfu.edu.cn (H.F.); fangyq0806@163.com (Y.F.); yang-5326@swfu.edu.cn (X.Y.); lr15007037606@swfu.edu.cn (Y.Z.); hzr1477991608@163.com (Z.H.); mulan1016@163.com (L.M.); lijuan@swfu.edu.cn (J.L.); 2 State Key Laboratory of Plant Diversity and Specialty Crops, Xishuangbanna Tropical Botanical Garden, Chinese Academy of Sciences, Kunming 650223, China; jhchen@xtbg.ac.cn; 3 Panzhihua City West District Ecological Environment Monitoring Station, Panzhihua 617000, China; qiyi_chen71@outlook.com

**Keywords:** chickpea, *ARF* gene family, *MONOPTEROS/ARF5*, pinnate compound leaf

## Abstract

Leaf architecture critically impacts crop yield. The Auxin Response Factor (*ARF*) family is a key regulator of leaf development, yet remains uncharacterized in the important legume crop chickpea (*Cicer arietinum* L.), which bears pinnate compound leaves. Here, we performed a genome-wide identification and analysis of *ARF* genes in chickpea. We identified 33 *CaARF* genes and resolved their phylogenetic structure through comparison with six other key dicot species. The analysis revealed a deeply conserved core set of ARF proteins across species, all sharing the N-terminal DNA-binding domain (DBD), with most the C-terminal PB1 domain, connected by a middle region (MR). We also uncovered instances of lineage-specific expansion, e.g., a chickpea-specific *ARF* clade, which is characterized by the absence of the C-terminal PB1 domain. Expression profiling using public transcriptome data and qRT-PCR revealed distinct spatiotemporal expression patterns for *CaARF* genes across tissues and during compound leaf development. Detailed in situ hybridization analysis for selected candidates, chosen based on phylogenetic proximity to known leaf-development-related *ARFs* in other species, localized their transcripts to specific regions within compound leaf primordia. Focusing on *CaARF5*, the closest ortholog of *Arabidopsis MONOPTEROS/ARF5*, we confirmed its nuclear localization and dynamic expression during chickpea leaf development. Functional complementation assays demonstrated that *CaARF5* could restore developmental defects in the *Arabidopsis mp* mutant. Our study establishes an evolutionary and molecular framework for the chickpea *ARF* family, highlighting conserved features and species-specific innovations, and provides essential resources for future research on auxin-mediated leaf development and *ARF*-targeted legume breeding.

## 1. Introduction

Chickpea (*Cicer arietinum* L.) is one of the most important legume crops globally, valued as a rich source of protein, carbohydrates, minerals, vitamins, and health-promoting fatty acids. It serves as a primary source of plant-based protein and forage, particularly in arid and semi-arid regions [1]. Beyond its primary harvest as a dry pulse, the chickpea holds significant value as a vegetable crop: in many regions, the immature green seeds and pods are widely consumed as a nutritious vegetable, featuring in diverse culinary preparations such as salads, sautés, and stews [2]. A defining morphological trait of the chickpea plant is its pinnate compound leaf, whose architecture directly influences photosynthetic efficiency and yield potential [3]. The morphogenesis of this compound structure—a process involving the precise initiation, patterning, and outgrowth of multiple leaflets from a common primordium—represents a sophisticated developmental program [4]. Unraveling the molecular networks governing this process is not only a fascinating question in plant developmental biology but also holds substantial promise for targeted genetic improvement aimed at optimizing canopy architecture and enhancing light capture efficiency.

The phytohormone auxin acts as a master regulator of plant growth and organogenesis, with its transcriptional outputs largely mediated by a family of plant-specific transcription factors known as Auxin Response Factors (*ARFs*) [5]. ARF proteins function as the executive arm of the auxin pathway by directly binding to Auxin Response Elements (AuxREs, typically TGTCNN) in the promoters of target genes, thereby activating or repressing their transcription to coordinate developmental processes [6,7,8]. Canonical ARF proteins are modular, comprising three primary domains: an N-terminal B3-type DNA-binding domain (DBD), a highly variable middle region (MR) that often dictates transcriptional activity (glutamine-rich for activators, serine/proline-rich for repressors), and a conserved C-terminal domain (CTD), now recognized as a Phox and Bem1 (PB1) domain that mediates protein–protein interactions, notably with Aux/IAA repressors [5,9,10]. In the prevailing model of nuclear auxin pathway (NAP), auxin promotes the degradation of Aux/IAA proteins, releasing ARFs from repression and allowing them to modulate downstream gene networks [11,12]. Phylogenetic and functional studies indicate that *ARFs* have diverged into three functionally different classes: A, B and C. Class A *ARFs* (A-*ARFs*) are primarily regulated by auxin through the NAP and acting as transcriptional activators. In contrast, B- and C-*ARFs* are considered transcriptional repressors [13]. Extensive research across model plants like *Arabidopsis thaliana* has elucidated specialized biological roles for specific *ARFs*: A-*ARF5/MONOPTEROS* regulates embryo patterning and vascular development [14,15]; A-*ARF7* and A-*ARF19* coordinate lateral root formation and tropisms [16,17]; B-*ARF3/ETTIN* and B-*ARF4* govern abaxial-adaxial identity in lateral organs [18]; *ARF6* and *ARF8* integrate hormonal signals during reproductive development [19,20,21]; *ARF17* coordinates the development of different sporophytic cell layers in anthers [22]. Beyond *Arabidopsis*, *ARFs* in crops such as rice, maize, tomato, and cotton have been implicated in stress responses, nutrient homeostasis, inflorescence structure, fruit set, and fiber development, underscoring their functional versatility and agricultural relevance [23,24,25,26,27,28,29].

Previous studies had shown that multiple *ARF* members regulate different aspects of leaf developmental processes. In *Arabidopsis*, *ARF5/MP* serves as a master regulator during early leaf development. Its functions are multifaceted: it is indispensable for leaf initiation, contributes to the establishment of leaf dorsoventral (adaxial-abaxial) polarity, and activates downstream genes such as *WUSCHEL-RELATED HOMEOBOX 1* (*WOX1*) and *PRESSED FLOWER* (*PRS*) to promote lamina outgrowth and flattening [30]. Other A-*ARFs*, such as *ARF7* and *ARF19*, are known to cooperatively influence leaf cell expansion [31,32]. In tomato, different A-*ARFs* (*SlMP*, *SlARF19a*, *SlARF19b*) contribute to leaflet formation with varying potency, largely correlated with their expression levels; the *slmp slarf19b* double mutant fails to initiate leaflet primordia, forming needle-like leaves [33]. In multiple species, B-*ARFs*, particularly *ARF3* and *ARF4* orthologs, are tightly integrated into core leaf developmental networks. *ARF3* and *ARF4* transcripts were specifically accumulated in the abaxial domains of developing leaf primordia. They collaborate with factors like *KANADI* to establish and maintain leaf polarity [18]. In *Medicago truncatula*, adaxially expressed ARGONAUTE7 (*MtAGO7*) facilitates ta-siRNA biosynthesis to negatively regulate the abaxially enriched *MtARF3* [34]. The plants overexpressing a *TAS3* ta-siRNA-insensitive mutant of *MtARF3* (*MtARF3m*) exhibits increased leaflet number [35]. In tomato *wiry* mutant (*slago7*), reduced ta-siRNA levels and *SlARF3/4* accumulation lead to fewer, nearly radial leaflets, and occasional needle-like leaves, whereas reducing *SlARF3* and *SlARF4* levels can rescue the wiry leaf lamina, and increased activity of either can phenocopy wiry leaves [36]. In summary, leaf morphogenesis is finely tuned by a complex network of interactions mediated by *ARFs* across different classes, integrating transcriptional activation, repression, and small RNA-mediated post-transcriptional regulation.

In this study, we systematically identified and characterized the *ARF* gene family in chickpea, with a focus on their potential roles in the development of the species’ characteristic odd-pinnate compound leaves. A total of 33 *CaARF* genes were identified from the genome. Subsequent comprehensive analyses—including phylogenetics, protein domain architecture, gene structure, chromosomal distribution, duplication events, and promoter cis-element analysis—defined the family’s genomic organization and potential regulatory features. Expression profiling via transcriptomics and qRT-PCR revealed that several class A and class B *CaARF* genes are highly expressed in the shoot apical meristem and young leaf primordia, implicating them in early leaf morphogenesis. Functional analysis focused on *CaARF5*, which showed specific accumulation in early leaf primordia and partially rescued the developmental defects of the *Arabidopsis mp* mutant, demonstrating functional conservation. While *ARF* families have been described in some other legumes [37,38,39], a dedicated analysis in chickpea was previously absent. Our work fills this gap and provides a multifaceted resource that integrates evolutionary, molecular, and expression data. This foundation establishes the chickpea *ARF* family as a key system for probing the mechanisms of auxin-mediated leaf development and identifies potential targets for legume improvement strategies.

## 2. Results

### 2.1. Identification of 33 Members of ARF Family Genes in Chickpea

Based on genome-wide analysis, a total of 33 *ARF* family members were identified in the chickpea genome (cv. CDC Frontier, version v2.0.a1), which were sequentially designated as *CaARF1* to *CaARF32* primarily according to their phylogenetic relationships with *Arabidopsis* ARF proteins (Supplemental Table S2). Prediction of physicochemical properties revealed substantial variation in the protein lengths, ranging from 132 (CaARF32) to 1125 amino acids (CaARF19c), corresponding to a molecular weight range of approximately 14.76 to 126.33 kDa. The isoelectric point (pI) values ranged from 5.55 (CaARF5) to 10.22 (CaARF29). The majority of the family members (26/33) had a theoretical pI below 7.0, indicating that the *CaARF* family is predominantly acidic; only seven proteins (CaARF9b, CaARF10a, CaARF10b, CaARF16a, CaARF16b, CaARF16c, and CaARF29) displayed basic characteristics. The grand average of hydropathicity (GRAVY) was negative for 31 members, suggesting an overall hydrophilic nature. The two exceptions, CaARF31 and CaARF32, had positive GRAVY values. In terms of instability, all members exhibited instability index values exceeding 40, indicating that the *CaARF* family proteins are unstable. The aliphatic index ranged from 65.43 to 107.05, with CaARF31 and CaARF32 surpassing 100, implying that these two proteins may possess relatively higher thermal and structural stability (Supplemental Table S3).

In summary, the chickpea *CaARF* family exhibits substantial diversity in key physicochemical properties, including length, molecular weight, and charge, while being collectively characterized by acidic pI, hydrophilicity, and predicted instability.

### 2.2. Phylogenetic Analysis and Classification of the ARF Family in Chickpea

**Figure 1:**
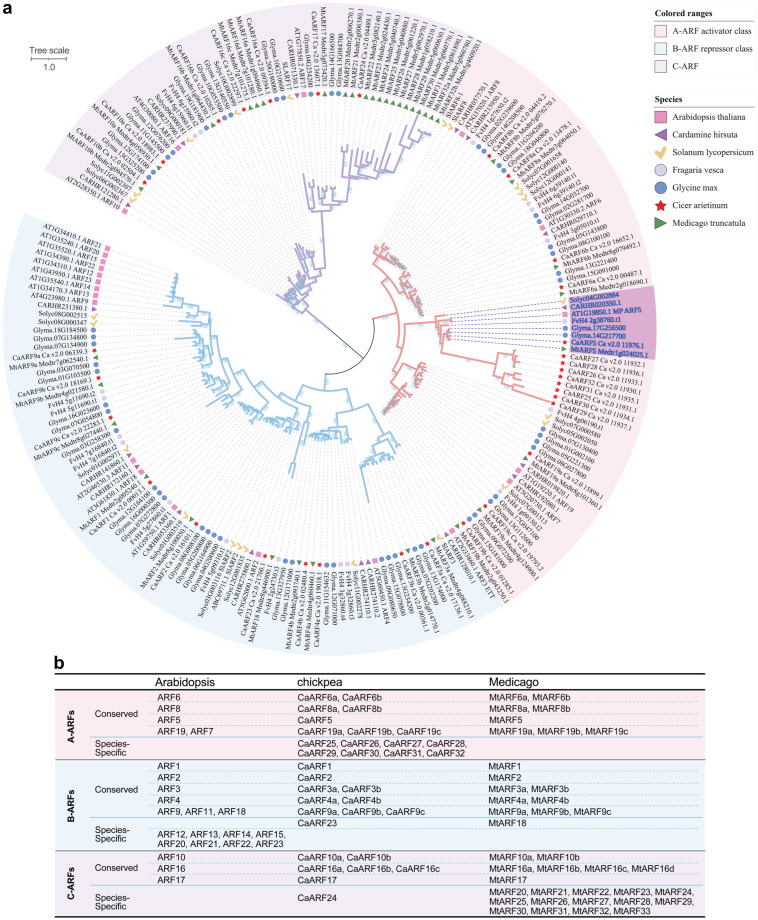
Phylogenetic analysis of the ARF protein family in chickpea and other plant species. (**a**) Phylogenetic tree constructed using ARF protein sequences from chickpea (*Cicer arietinum*) and six representative species: *Arabidopsis thaliana*, *Glycine max*, *Medicago truncatula*, *Fragaria vesca*, *Solanum lycopersicum*, and *Cardamine hirsuta*. the corresponding bootstrap values are indicated at the branch nodes, and the branch length scale bar represents 1.0. The dark blue font indicates the *ARF5* homologous gene. The three major evolutionary subfamilies are indicated by distinct colored regions: A-*ARF* (Pink), B-*ARF* (Blue), and C-*ARF* (Purple). (**b**) Statistical distribution of member numbers within the A-*ARF*, B-*ARF*, and C-*ARF* subfamilies in *Arabidopsis thaliana*, *Cicer arietinum*, and *Medicago truncatula*.

To elucidate the evolutionary relationships of the chickpea *ARF* family, we performed a phylogenetic analysis using ARF protein sequences from chickpea, *Medicago truncatula*, soybean (*Glycine max*), *Arabidopsis thaliana*, and three other compound-leafed species—*Fragaria vesca*, *Solanum lycopersicum*, and *Cardamine hirsuta*. The resulting midpoint-rooted tree resolves all ARF proteins into three major, well-supported subfamilies, corresponding to the A-*ARFs*, B-*ARFs*, and C-*ARFs* (Figure 1a). Each subfamily comprises multiple distinct branches that predominantly consist of orthologous proteins from the seven species analyzed. Statistical analysis of orthologous groups revealed that, compared to *Arabidopsis*, certain branches contain a notably larger number of members in chickpea and other legumes. For instance, the orthologous groups containing *AtARF9*, *AtARF16*, and *AtARF19* are significantly expanded in legumes, suggesting lineage-specific gene family expansion events during legume genome evolution (Figure 1b).

We also identified a limited number of species-specific *ARF* branches belonging to different subfamilies. Chickpea possesses a specific clade within the A-*ARF* subfamily that is phylogenetically most closely related to the *ARF5* clade, while *Arabidopsis thaliana* and *Medicago truncatula* each possesses a distinct, species-specific clade within the B-*ARF* and C-*ARF* subfamilies, respectively (Figure 1b). These unique clades likely reflect functional divergence arising from distinct biological adaptations in each species. Collectively, the phylogenetic framework established here provides a crucial foundation for elucidating functional diversification and molecular evolution of the *ARF* gene family in chickpea.

### 2.3. Gene Structure and Conserved Domain Analysis of the ARF Family in Chickpea

**Figure 2:**
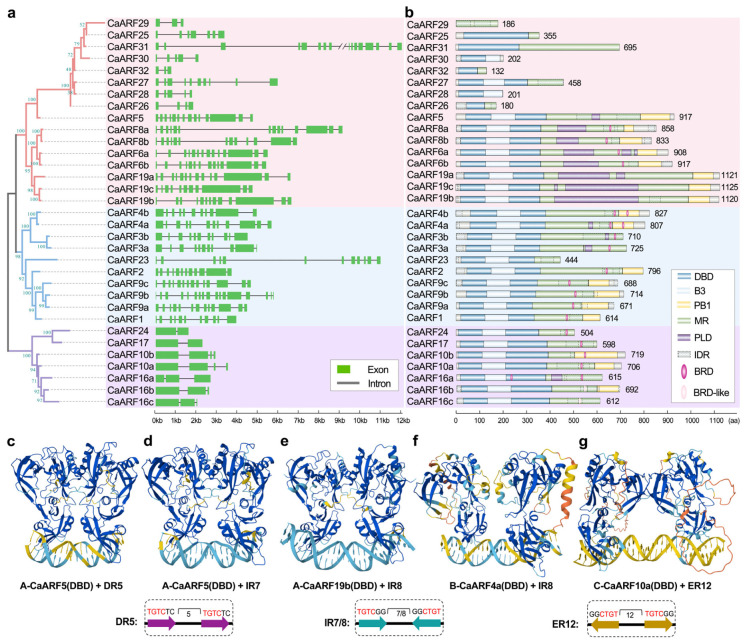
Analysis of Gene Structure and Protein Conserved Domains in the Chickpea *ARF* Gene Family. (**a**) Gene structure of the chickpea *ARF* genes. Green rectangles represent exons, and introns are indicated by black lines. (**b**) Schematic diagram of ARF domains in chickpea. The number of amino acid residues for each ARF protein is labeled. DBD, DNA-binding domain; B3, B3 DNA-binding domain; PB1, protein–protein interaction domain; MR, middle region. PLD, prion-like domain. IDR, intrinsic disordered region; BRD, B3 repression domain. A scale bar is shown at the bottom of the chart. (**c**) A-CaARF5(DBD) binds to the inverted repeat DR5. (**d**) A-CaARF5(DBD) binds to the inverted repeat IR7. (**e**) A-CaARF19b(DBD) binds to the inverted repeat IR8. (**f**) B-CaARF4a(DBD) binds to the inverted repeat IR8. (**g**) C-CaARF10a(DBD) binds to the everted repeat ER12. In panels (c)–(g), the blue regions in the double helix structure indicate high confidence, while the yellow regions indicate medium confidence. Different colors within the dashed boxes represent distinct AuxRE sequences, specifically magenta for the DR5 motif, cyan for the IR7/8 motif, and orange for the ER12 motif.

Analysis of gene structures revealed that members within the same phylogenetic group generally share similar exon-intron patterns, with the notable exceptions of the chickpea-specific A-*ARF* clade members and *CaARF23*. Excluding these exceptions, members of the A and B-*ARF* subfamilies typically contain the higher number of exons (11–14), whereas C-*ARF* members possess significantly fewer (2–4). This pattern is consistent with the hypothesis that the A/B and C subfamilies diverged early in their evolutionary history (Figure 2a).

Domain architecture analysis shows that the majority of chickpea ARF members contain the canonical B3-type DNA-binding domain (DBD), Middle Region (MR), and C-terminal Phox and Bem1 (PB1) domain. However, the composition and presence of these domains exhibit significant divergence among subfamilies. Within the A-*ARF* subfamily, the eight members constituting the chickpea-specific clade have markedly shorter protein sequences and uniformly lack the PB1 domain. Their gene structures are also atypical and highly variable, suggesting these members may have diverged functionally from canonical ARF proteins. In contrast, the remaining A-*ARF* members outside this specific clade exhibit a complete domain architecture, comprising the DBD, B3, MR, and PB1 domains, and frequently contain PLD (Prion-like domain) and IDR (intrinsically disordered region) sequences (Figure 2b).

In the B-*ARF* subfamily, most members possess the DBD, B3, MR, and PB1 domains. However, the evolutionarily conserved pair *CaARF3a/3b* and the species-specific member *CaARF23* lack the PB1 domain. Within the C-*ARF* subfamily, four of the seven members lack the PB1 domain, while three retain it, indicating considerable variation in PB1 domain retention among C-*ARFs*. Additionally, a B3 repression domain (BRD, R/K/VLFG sequences) was found to be widely present in B- and C-*ARF* subfamily members, found in few A-*ARF* members (Figure 2b).

We employed AlphaFold3 to model the interaction between chickpea ARF DBDs and AuxREs, testing whether the clade-specific binding rules defined in *Arabidopsis* are conserved. Structural predictions for representative clade A (CaARF5, CaARF19b), B (CaARF4a), and C (CaARF10a) members confirm this conservation (Figure 2c–g). As anticipated, the clade A DBDs bind a broad spectrum of AuxRE configurations, the clade B DBD preferentially interacts with an IR8 motif, and the clade C DBD exhibits a preference for an ER13 motif. These models validate that the core principle of an *ARF*-AuxRE recognition code—where phylogeny dictates binding specificity—is maintained in chickpea, structurally defining the potential for divergent transcriptional outputs from a conserved *ARF* subfamily.

### 2.4. Chromosomal Localization and Synteny Analysis of CaARFs

**Figure 3:**
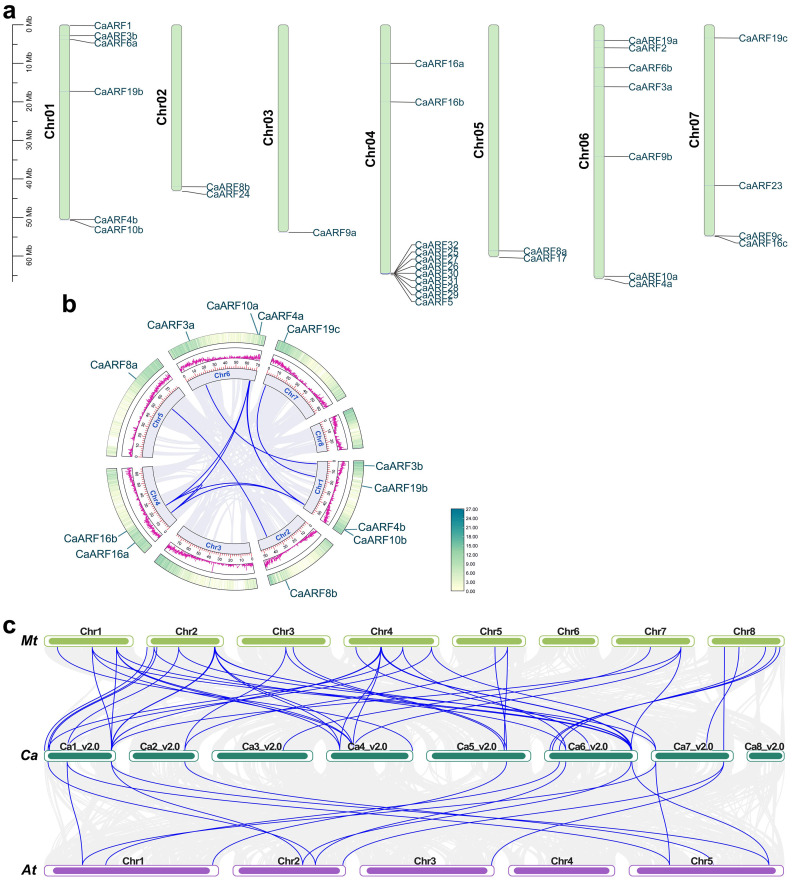
Chromosomal localization and synteny analysis. (**a**) Chromosomal localization of the *ARF* gene family in chickpea. The left scale indicates chromosome length (Mb), and the positions of *ARF* genes are marked on the right side of each chromosome. (**b**) Synteny analysis within the *ARF* gene family of chickpea. The middle circular magenta lines denote gene density. (**c**) Interspecific synteny of the *ARF* gene family between chickpea and model species.

Based on the chromosomal localization results of the chickpea (*Cicer arietinum*) *ARF* genes (Figure 3a; Supplemental Table S5), the *CaARF* family members exhibit a pronounced non-uniform distribution pattern across the chromosomes. Chromosome 4 harbors the highest number of *CaARF* genes. Notably, this includes three typical *ARF* members (*CaARF5*, *CaARF16a*, *CaARF16b*) and all members of the chickpea-specific clade, which together with *CaARF5* are arranged in a tight tandem array at the distal end of the chromosome, strongly suggesting evolution through recent tandem duplication events. Regarding the distribution pattern, certain genes, such as *CaARF19b*, *CaARF16b*, and *CaARF9b*, tend to be localized to the middle chromosomal regions or near the centromere, whereas the majority of the remaining genes are located on the distal regions of the chromosome arms.

To further explore their evolutionary relationships, we performed synteny analysis and identified a total of 10 segmental duplication pairs (Figure 3b; Supplemental Table S6), indicating that segmental duplication is one of the primary mechanisms driving the expansion of this gene family. The aforementioned uneven distribution and local clustering reflect that the *CaARF* family may have expanded through mechanisms such as segmental and tandem duplications.

Comparative synteny analysis revealed distinct evolutionary relationships between the chickpea *ARF* family and those of two reference species (Figure 3c). We identified 46 orthologous gene pairs between chickpea and the model legume *Medicago truncatula*. With the exception of the chickpea-specific clade members, nearly all core-set chickpea *ARF* genes have conserved orthologs in *M. truncatula*, indicating that the family’s expansion and genomic organization have been largely stable since the divergence of these legume lineages. In contrast, only 15 orthologous pairs were detected between chickpea and *Arabidopsis thaliana*. The maintenance of these corresponding genomic blocks across a greater evolutionary distance suggests they may have been under high selective pressure, preserving essential functions.

### 2.5. Cis-Acting Element Analysis of ARF Family Gene Promoters in Chickpea

**Figure 4:**
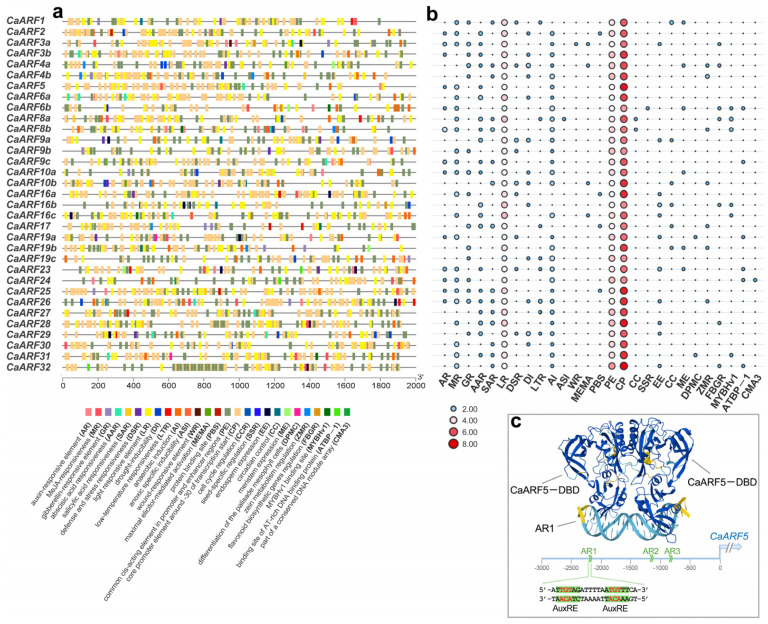
Analysis of cis-acting elements in the promoters of *CaARFs*. (**a**) The 2 kb upstream region from the start codon was analyzed using PlantCARE to identify promoter cis-acting elements, and the results were visualized with TBtools. Different colored boxes represent diverse promoter cis-acting elements. (**b**) Heatmap of promoter cis-acting element analysis for *ARF* genes in chickpea. AR: auxin-responsive element; MR: MeJA-responsiveness; GR: gibberellin-responsive element; AAR: abscisic acid responsiveness; SAR: salicylic acid responsiveness; DSR: defense and stress responsiveness; DI: drought-inducibility; LTR: low-temperature responsiveness; AI: anaerobic induction; ASI: anoxic-specific inducibility; WR: wound-responsive element; MEMA: maximal elicitor-mediated activation; LR: light responsive element; CCR: cell cycle regulation; SSR: seed-specific regulation; EE: endosperm expression; CC: circadian control; ME: meristem expression; DPMC: differentiation of the palisade mesophyll cells; ZMR: zein metabolism regulation; FBGR: flavonoid biosynthetic genes regulation; MYBHv1: MYBHv1 binding site; ATBP-1: binding site of AT-rich DNA binding protein; CMA3: part of a conserved DNA module array; PBS: protein binding site; PE: common cis-acting element in promoter and enhancer regions; CP: core promoter element around −30 of transcription start. (**c**) Predicted binding mode between the CaARF5 DBD and the AuxRE motifs in the target gene promoter. The upper panel illustrates the domain architecture of the CaARF5 protein (DBD and AR1–AR3 regulatory regions), while the lower panel depicts a 20 bp double-stranded DNA sequence containing two AuxRE motifs within the promoter region.

Analysis of cis-acting elements in the promoters of chickpea *ARF* genes revealed a complex regulatory architecture (Figure 4a,b; Supplemental Tables S8 and S9). The promoters are enriched not only with core promoter elements (e.g., TATA boxes) for precise transcriptional initiation but also with a diverse array of motifs associated with hormone response (including auxin), light signaling, and abiotic stress. This configuration suggests that *CaARF* genes function as integrator nodes, whose expression can be modulated by multiple internal and external signals. Notably, the promoters of several *CaARF* genes contain canonical Auxin Response Elements (AuxREs). Given the reported autoregulation of *Arabidopsis MP/ARF5*, we hypothesized a similar mechanism might exist in chickpea. We employed AlphaFold3 to predict the binding of the CaARF5 DNA-binding domain (DBD) to three potential AuxRE pairs (AR1–AR3) identified within its own promoter (Figure 4c). The structural model demonstrates high-confidence binding of the CaARF5-DBD to the AR1 motif, supporting the possibility that *CaARF5* can directly regulate its own transcription. This finding positions *CaARF5* not only as a key mediator of auxin signaling but also as a component subject to precise feedback regulation within the auxin response network.

### 2.6. Expression Analysis of CaARF Genes in Different Tissues

**Figure 5:**
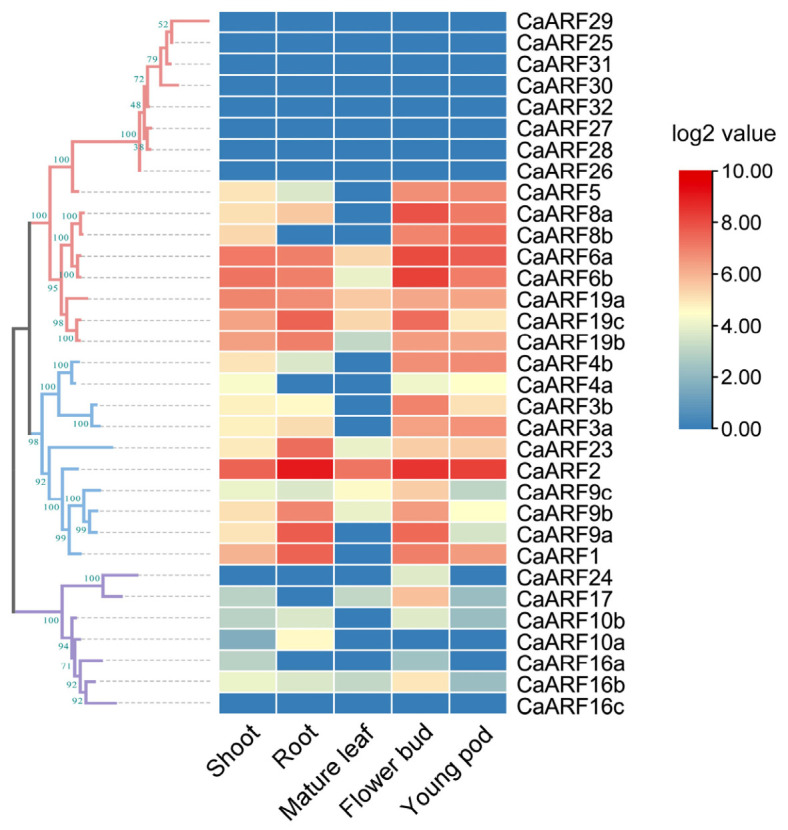
Expression profiles of the *CaARF* gene family in different tissues and organs. Normalized log2 transformed values were used with hierarchical clustering represented by the color scale (0–10). Blue indicates low expression, and red indicates high expression.

Heatmap analysis revealed distinct, subclass-specific expression profiles for the chickpea *CaARF* gene family across five tissues (Figure 5; Supplemental Table S10). Considerable heterogeneity was observed within the A-*ARF* subclass. Eight chickpea-specific A-*ARF* members (*CaARF25*-*32*) showed negligible expression in all tissues examined. In contrast, other A-*ARF* members exhibited enriched expression in young, developing tissues (shoot, flower bud, young pod) but uniformly low expression in mature leaves. Within the B-*ARF* subclass, most members displayed moderate, constitutive expression in young tissues, but markedly downregulated in mature leaves. A notable exception was *CaARF2*, which maintained high constitutive expression across all tissues. Members of the C-*ARF* subclass exhibited overall low or undetectable transcriptional abundance. These patterns suggest that A- and B-*ARF* members are likely key regulators of young organ development, while C-*ARF* members may be involved in distinct, possibly more subtle or condition-specific biological processes.

### 2.7. qRT-PCR Validation of Leaf Development-Associated CaARF Candidates

**Figure 6:**
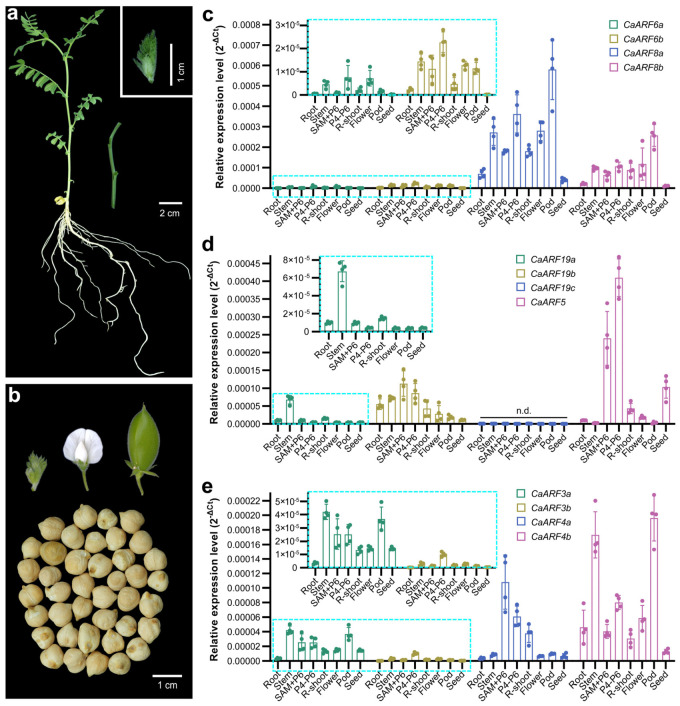
qRT-PCR validation of *CaARF* gene expression in different tissues and organs. (**a**) On the left is a chickpea plant at the vegetative stage; the middle shows the selected stem. Scale bar, 2 cm; the upper right corner indicates the selected vegetative shoot apex. Scale bar, 1 cm. (**b**) The upper panel shows, from left to right, the reproductive shoot apex, flower, and pod of chickpea; the lower panel shows seeds. Scale bar, 1 cm. (**c**) qRT-PCR validation of the expression levels of chickpea *CaARF6a*, *CaARF6b*, *CaARF8a*, and *CaARF8b* in different tissues. (**d**) qRT-PCR validation of the expression levels of chickpea *CaARF19a*, *CaARF19b*, *CaARF19c*, and *CaARF5* in different tissues. (**e**) qRT-PCR validation of the expression levels of chickpea *CaARF3a*, *CaARF3b*, *CaARF4a*, and *CaARF4b* in different tissues. The data are the mean ± SD from three biological replicates. (**c**–**e**) The blue dashed boxes highlight genes with a moderate or low expression level.

The development of the odd-pinnate compound leaf, a hallmark of chickpea, involves precise genetic regulation that remains poorly understood. Studies in model plants like *Arabidopsis* [31], tomato [33,36], and *Medicago truncatula* [34,35] have implicated specific A- and B-class *ARF* members in leaf morphogenesis. To identify chickpea *ARF* genes potentially involved in this process, we analyzed their tissue-specific expression patterns (Figure 6a,b). Initial transcriptomic data provided a broad overview of *CaARF* expression across multiple organs. Based on this, we selected candidate genes from the A- and B-*ARF* subfamilies for detailed validation using quantitative reverse transcription PCR (qRT-PCR).

Within the A-*ARF* subclass, we first analyzed the expression of two closely related gene pairs: *CaARF6a/6b* and *CaARF8a/8b* (Figure 6c), because (i) they are orthologs of *Arabidopsis ARF6* and *ARF8*, two genes expressed in the petiole and vasculature of developing leaves [40]; and (ii) preliminary transcriptomic data revealed that these gene pairs show relatively high expression in young chickpea tissues (Figure 5). A clear hierarchy in overall expression abundance was observed between the pairs: *CaARF8a* and *CaARF8b* exhibited substantially higher transcript levels across all examined tissues compared to *CaARF6a* and *CaARF6b*, with *CaARF6b* being barely detectable. At the level of tissue specificity, all four genes showed preferential expression in young, developing tissues. Notably, *CaARF8a/8b* showed particularly high expression in pods.

Given the critical role of *ARF5* and *ARF19* orthologs in tomato leaflet initiation [33,36], we examined the expression of their chickpea counterparts: *CaARF5*, *CaARF19a*, *CaARF19b*, and *CaARF19c* (Figure 6d). *CaARF5* was the most highly expressed, followed by *CaARF19b*. *CaARF19a* showed moderate expression, while *CaARF19c* was undetectable in all tissues tested. Their tissue-specific expression patterns were also divergent. *CaARF5* was most abundant in the shoots, young leaves, and seeds. *CaARF19b* displayed relatively high expression in the shoots, young leaves, stem, and root, whereas *CaARF19a* was specifically enriched in the stem.

Within the B-*ARF* subclass, we focused on *CaARF3a/3b* and *CaARF4a/4b* (Figure 6e), the orthologs of *Arabidopsis ARF3/ETT* and *ARF4*, which are known to participate in leaf adaxial-abaxial polarity establishment and leaf morphogenesis in *Arabidopsis*, and both pairs have undergone duplication in chickpea. Overall, *CaARF4a* and *CaARF4b* were consistently expressed at higher levels than *CaARF3a* and *CaARF3b* across most tissues. Examining tissue-specific profiles revealed further functional clues: *CaARF4b* expression was relatively high in pods and stems, while *CaARF4a* peaked in the shoots and young leaves. *CaARF3a* maintained a ubiquitous, low-to-moderate level of expression in all tissues except roots. *CaARF3b* was generally low except for a notable level in leaf primordia.

The qRT-PCR expression trends for the majority of genes validated and refined the patterns observed in the RNA-seq heatmap analysis. A notable exception was *CaARF19c*, which showed a signal in the heatmap but was undetectable by qRT-PCR. This discrepancy likely stems from fundamental methodological differences between the two techniques as well as potential variations in the biological samples used.

### 2.8. Spatiotemporal Expression Dynamics of Key CaARF Genes During Compound Leaf Morphogenesis

**Figure 7:**
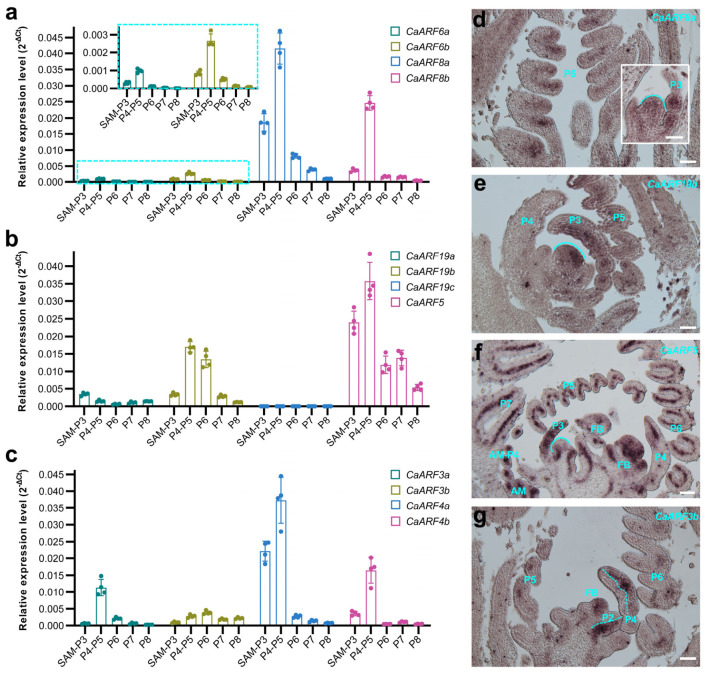
qRT-PCR validation of *CaARF* gene expression in different stages. (**a**) qRT-PCR validation of the expression levels of chickpea *CaARF6a*, *CaARF6b*, *CaARF8a*, and *CaARF8b* in shoot apical meristems at different developmental stages. The blue dashed boxes highlight genes with a moderate or low expression level. (**b**) qRT-PCR validation of the expression levels of chickpea *CaARF19a*, *CaARF19b*, *CaARF19c*, and *CaARF5* in shoot apical meristems at different developmental stages. (**c**) qRT-PCR validation of the expression levels of chickpea *CaARF3a*, *CaARF3b*, *CaARF4a*, and *CaARF4b* in shoot apical meristems at different developmental stages. The data are the mean ± SD from three biological replicates. (**d**–**g**) RNA in situ hybridization of *CaARF8a*, *CaARF19b*, *CaARF5* and *CaARF3b* mRNA in wild-type leaf primordia. Similar results were obtained from three independent experiments. P, plastochron. Scale bar, 50 μm.

To further delineate the roles of key *CaARF* genes in leaf development, we analyzed their expression patterns across a developmental series of leaf primordia via qRT-PCR. Consistent with our broader tissue survey, a comparison of expression levels among orthologous pairs revealed a conserved hierarchy: *CaARF8a* and *CaARF8b* were expressed at substantially higher levels than *CaARF6a* and *CaARF6b* (Figure 7a); *CaARF5* was the most highly expressed, followed by *CaARF19b*, with *CaARF19a* lower and *CaARF19c* nearly undetectable (Figure 7b); and *CaARF4a/4b* levels exceeded those of *CaARF3a/3b* (Figure 7c). Examining the temporal expression profile of each gene across developmental stages (SAM-P3, P4-P5, P6, P7, P8) revealed a common trend. Most genes exhibited their high transcript levels in the SAM-P3 stage, peaked during the P4-P5 leaf primordia stage, and subsequently declined as leaves underwent differentiation and maturation. Here, SAM denotes the shoot apical meristem, and P followed by a number (e.g., P3) refers to the leaf plastochron index, indicating successively older leaf primordia. This pattern indicates that these *ARFs* predominantly function in regulating morphogenetic events during organ initiation and patterning, rather than in maintaining the functions of mature tissues.

To complement the quantitative temporal data with spatial resolution, we performed in situ hybridization. Due to the high nucleotide sequence similarity (70–80%) among many homologous genes (e.g., *CaARF3b* with *CaARF3a/4a/4b*), we selected representative members from different *ARF* classes for analysis. *CaARF8a* transcripts were detected in the SAM and leaf primordia at various stages, showing a broad distribution throughout the P3 leaflet primordium that later became more restricted to the marginal region at P6 (Figure 7d). *CaARF19b* expression was observed at the SAM periphery and showed a strong signal in the adaxial domain of early leaf primordia (Figure 7e). Among all genes examined, *CaARF5* exhibited the strongest hybridization signal. It was detected in leaf primordia of all stages examined, floral primordia, and axillary meristems (Figure 7f). Notably, within early leaf primordia, *CaARF5* mRNA showed particularly prominent and specific accumulation in the region of provascular tissue initiation. This spatial expression pattern not only aligns with the qRT-PCR data showing high *CaARF5* expression during early development but also provides direct spatial evidence supporting a core regulator for *CaARF5* during leaf morphogenesis in chickpea. *CaARF3b* expression was detected in both leaf and floral primordia (Figure 7g). In early leaf primordia, transcripts were specifically enriched in the abaxial domain, a pattern analogous to the expression of its orthologs in *Arabidopsis* and *Medicago truncatula*, which are established regulators of abaxial cell fate and leaf patterning. Sense probe control experiments for the corresponding genes yielded no specific hybridization signals (Figure S1), confirming the specificity of the antisense probe signals.

Collectively, the distinct yet developmentally regulated spatial expression patterns of *CaARF8a*, *CaARF19b*, *CaARF5*, and *CaARF3b*—encompassing meristematic zones, specific leaf domains (adaxial, abaxial, marginal, vascular), and reproductive primordia—provide direct spatial evidence that the functions of these *ARF* genes are closely associated with fundamental processes in compound leaf development, including primordia initiation, polarity establishment, and tissue patterning.

### 2.9. In-Depth Characterization of CaARF5 Expression and Subcellular Localization of the Encoded Protein

Building on our preceding expression analyses, which highlighted *CaARF5* as a prominently expressed candidate during leaf development, we hypothesized that it plays a central regulatory role. To rigorously test this hypothesis and delineate its functional context, we conducted a multi-faceted characterization of *CaARF5*.

**Figure 8:**
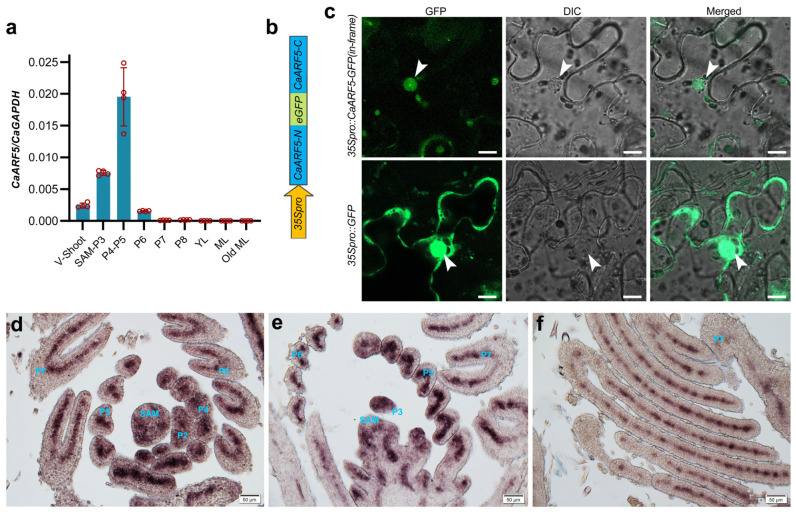
Spatiotemporal expression pattern analysis of the *CaARF5* gene. (**a**) Transcription signals of *CaARF5* were detected in different tissues and developing shoot apices by real-time quantitative PCR. V-shoots: vegetative shoot apical meristem; SAM: shoot apical meristem; YL: young leaf; ML: mature leaf; Old ML: old leaf. The data are the mean ± SD from three biological replicates. (**b**) Schematic diagram of the construction of the *35Spro::CaARF5-GFP*(in-frame) fusion expression vector. (**c**) Subcellular localization of CaARF5 in tobacco leaf epidermal cells. Arrows point to cell nuclei. Scale bar, 10 μm. (**d**–**f**) In situ hybridization analysis of *CaARF5* in the shoot apical meristem of wild-type chickpea. Longitudinal sections of the shoot apex were prepared using the paraffin sectioning method. P, plastochron. Scale bar, 50 μm.

We first analyzed its transcript levels in leaf primordia at different developmental stages using qRT-PCR. The results showed that *CaARF5* was highly expressed in the shoot apical meristem (SAM), with particularly significant enrichment in the early leaf developmental stages (P4–P5 leaf primordia), suggesting that it may play a regulatory role in early organogenesis, participating in axial growth and vascular development (Figure 8a). As leaf development progressed to the P6–P8 stages, its expression gradually declined and was maintained at relatively low levels in mature leaves (ML) and old mature leaves (Old ML).

To determine the subcellular compartment in which CaARF5 functions, a fusion construct *35Spro::CaARF5-GFP*(in-frame) (Figure 8b) was transiently expressed in tobacco epidermal cells. Confocal microscopy showed that the CaARF5-GFP(in-frame) fluorescence was exclusively localized to the nucleus, as delineated in the DIC image, in contrast to the diffuse nucleus-cytoplasmic distribution of free GFP, confirming CaARF5 as a nuclear protein (Figure 8c).

Finally, we performed high-resolution RNA in situ hybridization to map the spatial distribution of *CaARF5* mRNA during leaf development (Figure 8d–f). Transcripts were specifically detected in the SAM and leaf primordia. From stages P2 to P4, expression was broad and strong throughout the early compound leaf common primordium, including incipient leaflet and stipule domains. By stage P5, expression became more restricted to the central region of the leaflet primordia. As development proceeded to stages P6 and P7, hybridization signals were prominently associated with the developing vascular bundles, coinciding with vasculature differentiation. Together, these data delineate a dynamic and stage-specific expression pattern for *CaARF5*, underscoring its potential involvement in multiple key events during compound leaf morphogenesis, from primordia initiation to vascular patterning.

### 2.10. Functional Conservation Analysis of Chickpea CaARF5 in Arabidopsis

**Figure 9:**
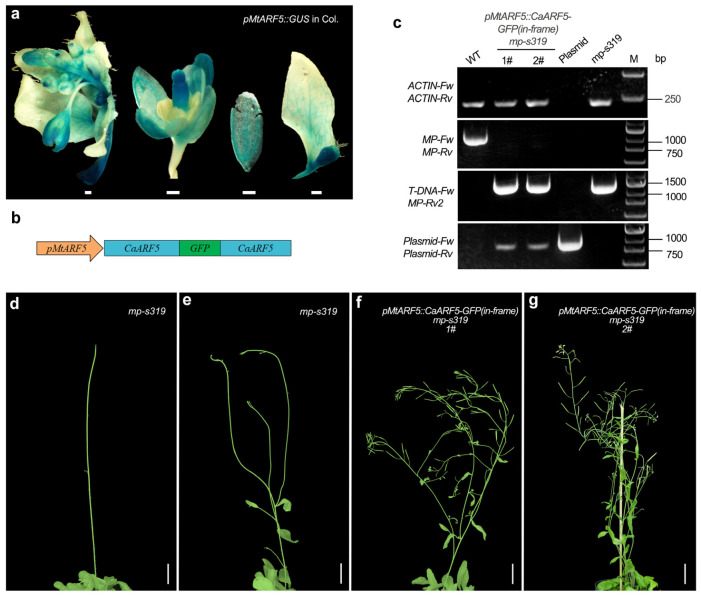
Phenotypic traits of ectopically overexpressed *CaARF5* in transgenic *Arabidopsis*. (**a**) Staining results of positive *Arabidopsis* wild-type Col plants transformed with *pMtARF5::GUS*. Scale bar, 200 μm. (**b**) Schematic diagram of the *pMtARF5::CaARF5-GFP*(in-frame) vector; (**c**) PCR genotyping of transgenic plants expressing *pMARF5::CaARF5-GFP*(in-frame) (in the *mp-s319* background). M, DNA molecular weight marker (bp); wild-type (WT), the recombinant plasmid (Plasmid), and genomic DNA from the *mp-s319* mutant were used as controls. (**d**,**e**) *Arabidopsis mp-s319* homozygous mutant lines; Scale bar, 1 cm. (**f**,**g**) *Arabidopsis* transgenic lines 1# and 2#. Scale bar, 1 cm.

To assess the functional conservation of the chickpea *CaARF5* gene, we conducted a heterologous complementation assay in *Arabidopsis*. We first confirmed the activity of a promoter from the *Medicago truncatula ARF5* ortholog (*pMtARF5*), in *Arabidopsis*. Histochemical staining revealed that a *pMtARF5::GUS* reporter was specifically expressed in reproductive and vegetative tissues, including inflorescences, stems, floral buds, and developing leaves, with signal enrichment in the vascular region of leaf primordia (Figure 9a).

We then constructed a fusion vector, *pMtARF5::CaARF5-GFP*(in-frame) (Figure 9b), and introduced it into heterozygous *mp-s319* mutants. Transgenic lines were selected by Basta resistance and validated by PCR genotyping (Figure 9c). Compared to the severe developmental defects of the *mp-s319* homozygous mutant—which forms a pin-like inflorescence and is completely sterile (Figure 9d,e)—the complemented lines expressing *pMtARF5::CaARF5-GFP*(in-frame) developed normal inflorescences and exhibited a partial restoration of fertility (Figure 9f,g). These results demonstrate that *CaARF5* can effectively rescue the reproductive defects of the *Arabidopsis mp* mutant, indicating a high degree of functional conservation.

## 3. Discussion

Chickpea is an important dual-purpose crop globally used for both food and feed, and its leaf shape directly affects photosynthetic efficiency and yield. Auxin Response Factors (ARFs) are key transcriptional regulators that interpret auxin gradients to direct developmental outcomes, including the morphogenesis of complex structures like compound leaves [41]. A previous study by Die et al. [42] first identified the ARF family in chickpea, reporting 24 members based on an earlier genome assembly (Kabuli type, CDC Frontier) and employing a nomenclature system according to chromosomal location. Our study provides substantial updates and advancements. We employed the widely accepted, phylogeny-based classification (A, B, and C clades) anchored to *Arabidopsis* orthologs, enabling direct functional inference and meaningful cross-species evolutionary comparisons. Furthermore, utilizing an improved, more contiguous reference genome integrated with pan-genome resources, coupled with refined Hidden Markov Model (HMM) profiles and a systematic screening strategy, we identified 33 ARF family members. The increase in the number of identified members stems from several key improvements: (1) the use of a more complete and accurately annotated genome assembly, which recovers loci missed in earlier versions; (2) the application of updated, more sensitive HMMs and adjusted significance thresholds; and (3) the adoption of inclusive verification criteria that retained putative ARFs lacking canonical domains (e.g., the C-terminal PB1 domain), with subsequent phylogenetic analysis used for validation. This approach ensures a more comprehensive and accurate catalog. Collinearity analysis indicated that segmental duplication is the primary mechanism driving the expansion of the chickpea *ARF* family, consistent with the expansion patterns of *ARF* genes in soybean and common bean [43]. Notably, the chickpea *ARF* genes are unevenly distributed across chromosomes, forming a local gene cluster on chromosome 4, suggesting that tandem duplication may have also played a role in the evolution of this family.

The discovery of a chickpea-specific *ARF* clade presents an intriguing paradox. While its members, which lack the canonical C-terminal PB1 domain and are arranged in tandem with *CaARF5*, evidently originated from a recent duplication event, they are consistently expressed at barely detectable levels. This minimal expression suggests these genes are unlikely to perform crucial functions in routine development, though a role in specific contexts or as subtle modulators of auxin responses remains possible. The very persistence of this low-expressing, truncated clade underscores the remarkable evolutionary constraint on the *CaARF5* locus. The strong selective pressure to maintain the low copy number and precise function of the core *CaARF5* gene may have tolerated the retention of these duplicates only under strict regulatory silencing, highlighting the fundamental importance of *ARF5* dosage and protein structure.

*ARF5* is one of the most thoroughly studied members of the *ARF* family. Over more than three decades of research, scientists have established that *MP/ARF5* plays core regulatory roles in multiple developmental processes in *Arabidopsis*, including embryogenesis, vascular differentiation, leaf formation, and meristem maintenance [30]. In this study, we found that *CaARF5* is highly expressed in the shoot apical meristem and young leaf primordia of chickpea, and later becoming restricted to vascular bundles, with expression gradually decreasing as leaves mature. This spatiotemporal expression pattern is highly similar to that of *AtARF5* in *Arabidopsis* [30]. Importantly, *CaARF5* was able to partially rescue the severe developmental and fertility defects of the *Arabidopsis mp* mutant. This underscores the profound evolutionary constraint on the molecular function of this key auxin response regulator.

Mounting evidence indicates that the precision of *ARF*-mediated regulation depends, in part, on expression dosage. In tomato, for instance, the promotive strength of *A-ARFs* such as *SlMP* and *SlARF19* on leaflet production correlates directly with their expression levels [33]. These findings provide a relevant framework for understanding compound leaf development in chickpea. Our expression profiling of *CaARF* members revealed substantial variation in transcript abundance among paralogs within the same tissues, such as leaf primordia (Figure 6 and Figure 7). For example, closely related genes displayed a clear expression hierarchy: *CaARF5* > *CaARF19b* > *CaARF19a*, while *CaARF19c* was barely detectable. Similarly, *CaARF8a/8b* were expressed at significantly higher levels than *CaARF6a/6b*, and *CaARF4a/4b* showed consistently stronger expression across multiple tissues compared to their paralogs *CaARF3a/3b*. This differential expression pattern suggests that chickpea may fine-tune the intensity, spatiotemporal specificity, and responsiveness of auxin signaling output through a combination of gene duplication, structural divergence, and differential expression regulation.

Following duplication, paralogs often undergo subfunctionalization, where ancestral functions or expression domains are partitioned [44]. The expression hierarchy within the *CaARF5/19a/19b/19c* clade may represent a case of subfunctionalization. In *Medicago truncatula*, *MtARF2*/*MtARF3b/MtARF4a/MtARF4b* are post-transcriptionally regulated via the miR390/TAS3 ta-siRNA pathway to control lateral root development and nodule formation respectively, illustrating finely partitioned functional divergence among homologous genes [45]. Therefore, the differential expression hierarchy of paralogous genes in chickpea provides a molecular basis for the precise regulation of leaf morphology (e.g., leaflet number, incision depth) and a theoretical foundation for target gene selection and expression modulation in molecular breeding.

The DBD of ARF transcription factors recognizes AuxREs in gene promoters to directly activate or repress transcription. Previous studies have shown that *MP* can directly bind to and activate its own expression [30]. Our promoter cis-element analysis and structural modeling indicated that the upstream regions of many *CaARF* genes contain multiple AuxREs that could be efficiently bound by their own DBDs. For instance, the promoter of *CaARF5* itself contains an AuxRE pair predicted to be bound well by its DBD. This suggests that the expression of these *ARF* genes is subject to fine-tuning by their own or other ARF proteins. Therefore, our analysis provides a basis for a more comprehensive understanding of the precise spatiotemporal regulation of *ARF* genes during organ morphogenesis.

A central paradigm in legume compound leaf development is that *LFY/FLO* orthologs, such as *CaLFY* in chickpea, are crucial drivers of leaflet initiation. The spatiotemporal control of *LFY* activity, often through repression by specific factors, dictates the final leaf pattern [46]. In chickpea, the C2H2 zinc-finger protein MPL1, expressed basally, directly represses the distally enriched *CaLFY*, forming a complementary gradient that orchestrates the acropetal initiation of leaflets, maintaining the pinnate form. Notably, *mpl1* mutants exhibit upregulated expression of several *ARF* genes, including *CaARF5*, suggesting *MPL1* may also modulate the auxin pathway [4]. Studies in *Arabidopsis* flower development have established that the auxin response factor *ARF5/MONOPTEROS* directly activates *LFY* expression, indicating a core auxin–ARF–LFY regulatory axis [47]. Here, we found that several *CaARF* genes, including *CaARF5*, *CaARF8a/8b*, and *CaARF4a/4b*, exhibit strong and dynamic expression in early leaf primordia (P3-P5), coinciding with the critical window for patterning. This spatiotemporal overlap suggests a plausible model wherein auxin signaling, mediated by specific *CaARFs*, acts in concert with the MPL1-CaLFY module to jointly sculpt the compound leaf developmental blueprint.

Compound leaf development involves not only leaflet number determination but also the establishment of leaf polarity (adaxial-abaxial axis), a process in which auxin and *ARFs* are also key regulators. Our in situ hybridization analysis revealed the spatially specific expression of different *CaARF* genes in early primordia: *CaARF19b* transcripts were enriched in the adaxial domain, whereas *CaARF3b* was specifically localized to the abaxial domain. This pattern is conserved with orthologs in *Arabidopsis* and tomato. In Medicago, *MtARF3* expression is negatively regulated by *MtAGO7*-mediated *TAS3* ta-siRNA, and altering its level directly affects leaflet number [48]. The abaxial enrichment of *CaARF3b* observed here strongly suggests that a similar, small RNA-mediated post-transcriptional regulatory layer may exist in chickpea to finely calibrate *ARF* activity for leaflet morphogenesis.

In summary, this study establishes a foundational resource for the chickpea *ARF* family, delineating its evolutionary history, genomic organization, and expression landscape. We highlight both deep functional conservation, exemplified by *CaARF5*, and species-specific innovations, such as the PB1-lacking clade. The differential expression patterns among paralogs suggest a role for gene dosage in modulating auxin response outputs. The functional validation of *CaARF5* and the in silico evidence for its potential autoregulation provide a focused entry point for future mechanistic studies. This work not only advances our understanding of auxin-mediated leaf development in legumes but also identifies specific genetic components that could be targeted for molecular breeding strategies aimed at optimizing plant architecture in chickpea and related crops.

## 4. Materials and Methods

### 4.1. Searching for ARF Genes and Data Collection

Chickpea protein sequences were retrieved from the chickpea genome database (https://www.pulsedb.org/; accessed on 22 March 2026), and AtARF protein sequences were downloaded from The *Arabidopsis* Information Resource database (TAIR; https://www.arabidopsis.org/; accessed on 22 March 2026). To more comprehensively identify members of the chickpea *ARF* family, we employed two complementary search strategies. First, we performed an initial screening using the hidden Markov model (HMM) of the ARF characteristic domain obtained from the Pfam database (http://pfam.xfam.org/; accessed on 22 March 2026), via the simple HMM search function in TBtools (v2.376). Second, we conducted a BLASTP search against the chickpea proteome using AtARF sequences as queries with default parameters. The results from both search methods were merged to identify putative chickpea *ARF* homologs. Subsequently, all candidate protein sequences were verified for the presence of canonical ARF domains using the NCBI Conserved Domain Database (CDD; https://www.ncbi.nlm.nih.gov/Structure/cdd/cdd.shtml; accessed on 22 March 2026). The finalized set of chickpea ARF protein sequences was analyzed using TBtools (v2.376) to calculate their length, molecular weight, and isoelectric point.

### 4.2. Phylogenetic Analysis

The accession numbers or IDs of *ARF* homologs from *Arabidopsis*, tomato (*Solanum lycopersicum*), woodland strawberry (*Fragaria vesca*) and soybean were retrieved via BLASTp searches from Phytozome 14 (https://phytozome-next.jgi.doe.gov/; accessed on 24 March 2026). *ARF* homologs from *M. truncatula* and chickpea were identified through BLASTp searches in the Legume Information System (https://www.legumeinfo.org/; accessed on 24 March 2026). The phylogenetic tree was constructed using the maximum likelihood method in IQ-TREE v1.6.12 (http://www.iqtree.org/; accessed on 24 March 2026), with the JTT + F + G4 model, as suggested by the IQ-TREE model test tool (BIC criterion), using 1000 ultrafast bootstrap replicates and 5000 iterations. The resulting tree was then edited using MEGA 5.0 program and manually optimized for viewing clarity.

### 4.3. Gene Structures and Conserved Protein Domains

The genomic and coding sequences of *ARF* genes were obtained from chickpea genome database. Gene structures were visualized using the Gene Structure Display Server (GSDS) 2.0 (http://gsds.cbi.pku.edu.cn/; accessed on 27 March 2026). The output image was then refined using Adobe Photoshop for color enhancement and figure annotation, involving only cosmetic optimization without any alteration to the structural data. Conserved protein domains (DBD, B3 and PB1 domains) were identified using the NCBI BLAST suite. The core prion-like domain (PrD) was predicted with the Prion-like Amino Acid Composition (PLAAC) tool (http://plaac.wi.mit.edu/, Lcore = 60, α = 1; accessed on 27 March 2026). All protein domains were visualized using IBS 2.0 (https://ibs.renlab.org/#/server; accessed on 27 March 2026).

### 4.4. Protein–DNA Complex Structure Prediction

The three-dimensional structures of the DNA-binding domains (DBDs) from CaARF5, CaARF19b, CaARF4a, and CaARF10a in complex with a pair of AuxRE motifs (TGTCGG) (https://alphafoldserver.com/; accessed on 28 March 2026), were predicted using AlphaFold3 with default parameters [49].

### 4.5. Chromosome Locations, Syntenic Analysis and Cis-Elements Analysis of CaARFs

Chromosomal distribution information of *ARF* genes was obtained based on the genome GFF3 file. The chromosomal locations were visualized using TBtools software (v2.376). Duplication events of *CaARF* genes were detected by calling the MCScanX tool through TBtools, and segmental duplications were visualized using the Advanced Circos function. The One Step MCScanX tool was employed, together with genome annotation and sequence files, to predict syntenic relationships of *ARF* genes among chickpea, *M. truncatula*, and *A. thaliana*. The promoter sequences spanning 2000 bp upstream of the start codon of 33 *CaARF* genes were submitted to the PlantCARE database (http://bioinformatics.psb.ugent.be/webtools/plantcare/html/; accessed on 30 March 2026), for cis-acting element prediction, and the physical distribution of these elements was visualized using TBtools software (v2.376).

### 4.6. Plant Materials

The wild-type chickpea used in this study was CDC Frontier (Kabuli type), obtained from the USDA Western Regional Plant Introduction Station (Pullman, WA, USA) via Dr. Clarice Coyne. *A. thaliana* accession Col. and *M. truncatula* cv. R108 served as the wild-type references. Seeds of the *Arabidopsis mp-s319* mutant were kindly provided by Professor Zhao Zhong (University of Science and Technology of China, Hefei, China). All plants were grown in greenhouses under the following controlled conditions: a relative humidity of 50–60%, a temperature range of 24 °C during the day and 20 °C at night, a light intensity of 150 μmol/m^2^/s, and a 16 h light/8 h dark cycle.

### 4.7. RNA Extraction and Real-Time qPCR Analysis

Total RNA was isolated from samples using the RNAEx TriZol solution (GK3006, GENEray Biotech, Shanghai, China) according to the manufacturer’s protocol. First-strand cDNA was synthesized from 2 μg of total RNA using the HiScript II 1st Strand cDNA Synthesis Kit (R211, Vazyme, Nanjing, China). The resulting cDNA was diluted to a final volume of 100 μL with RNase-free water for subsequent analysis. Quantitative real-time PCR (real-time qPCR) was performed in 15 μL reaction volumes on a LightCycler 480 system (Roche, Basel, Switzerland). Each reaction contained 2.5 μL diluted cDNA, 0.45 μL of each gene-specific primer (10 μM), 7.5 μL of 2 × QuantFast Green qPCR SuperMix (M2211, Magic-bio, Hangzhou, China), and 4.1 μL of RNase-free water. All reactions were performed in triplicate. The thermal cycling conditions were as follows: 95 °C for 3 min; followed by 40 cycles of 95 °C for 10 s, 58 °C for 10 s and 72 °C for 20 s. The housekeeping gene *CaGAPDH* was used as internal reference for normalization. Relative gene expression levels were calculated using the 2^−ΔΔCT^ method [50]. All primers used for real-time qPCR in this study are listed in Supplemental Table S1. The expression profiles of *CaARF* genes in different tissues were predicted using the CTDB website (http://223.31.159.7/ctdb/; accessed on 3 April 2026), and visualized with TBtools software (v2.376).

### 4.8. RNA In Situ Hybridization

In situ hybridization was performed as previously described [50,51,52], with modifications for chickpea tissues. Briefly, shoot apices were harvested from 2-week-old and 6-week-old greenhouse-grown wild-type (cv. CDC Frontier) plants. Samples were immediately fixed in FAA fixative, processed overnight using an automated tissue processor (ASP200S, Leica, Wetzlar, Germany), and embedded in paraffin with a heated embedding station (EG1150, Leica, Wetzlar, Germany).

Digoxigenin (DIG)-labelled antisense RNA probes were prepared using the DIG RNA Labeling Kit (11175025910, Roche, Basel, Switzerland). The full length coding sequences of *CaARF8a*, *CaARF19b*, *CaARF5* and *CaARF3b* were amplified with the reverse primer (for antisense probe) or the forward primer (for sense probe) containing a T7 promoter sequence (5′-TGTAATACGACTCACTATAGGGC-3′) at its 5′end. The resulting amplicons served as templates for in vitro transcription to generate DIG-labeled RNA probes, which were subsequently hydrolyzed to fragments of 100–200 bp.

Paraffin-embedded shoot apices were sectioned into 8-μm-thick sections using a microtome (RM2235, Leica, Wetzlar, Germany). Sections were deparaffinized, rehydrated, and treated with 1 mg/L Proteinase K (03115879001, Roche, Basel, Switzerland) for 30 min at 37 °C. Following a brief post-fixation in FAA for 10 min and dehydration through an ethanol series, sections were hybridized with the DIG-labeled probes overnight at 55 °C. Post-hybridization, signals were detected by incubation with Anti-DIG-AP Fab fragments (11093274910, Roche, Basel, Switzerland) for 90 min, followed by colorimetric development with NBT/BCIP substrate (11681451001, Roche, Basel, Switzerland) for 48 h–72 h. Images were captured using a light microscope (BX63, Olympus, Tokyo, Japan). All primer sequences used for probe synthesis are listed in Supplemental Table S1.

### 4.9. Subcellular Localization

Subcellular localization was analyzed via transient expression in tobacco leaves. *Agrobacterium tumefaciens* strain EHA105 harboring the target vector was cultured overnight in a shaker at 28°C. The next day, 50 ul of the bacterial culture was added to 5 mL of fresh LB liquid medium and incubated at 28 °C with shaking until an OD600 of 1.0–1.5 was reached. The culture was centrifuged at 3900 rpm for 10 min, and the supernatant was discarded. During centrifugation, MMA buffer (10 mM MES pH 5.6, 10 mM MgCl_2_, 200 μM acetosyringone) was prepared. The bacterial pellet was resuspended in MMA buffer to an OD600 of 1.0 and incubated at room temperature for 1 h. Fully expanded tobacco leaves were selected, and small wounds were gently made on the abaxial surface using a needle. The bacterial suspension was infiltrated into the wounds using a 1 mL syringe without a needle. The plants were incubated in the dark at 28 °C for 36–48 h. The lower epidermal peel of the leaf was then transferred to a clean coverslip, mounted, and observed under a laser confocal microscope. GFP signals were detected under 488 nm excitation light.

### 4.10. GUS Staining

GUS staining was performed as previously described [53], with modifications for Arabidopsis tissues. The brief procedure is as follows. First, the samples were fixed: fresh tissues were placed in centrifuge tubes, covered with 90% acetone, and incubated on ice for 30 min, followed by rinsing twice with phosphate buffer. Next, for staining: after fixation, the samples were immersed in GUS staining solution, vacuum-infiltrated for 30 min, and incubated overnight at 37 °C. Finally, after destaining with 75% ethanol, the samples were imaged under a stereomicroscope.

### 4.11. Vector Construction and Plant Transformation

A GFP reporter construct was generated by in-frame fusion of GFP to the *CaARF5* coding sequence between residues Ile492 and Lys493. The N- and C-terminal fragments of *CaARF5* and the full-length GFP were amplified separately and assembled by overlap-extension PCR. The resulting fusion fragment was ligated into the binary vector pCAMBIA3301, creating a CaMV35S promoter-driven CaARF5-GFP(in-frame) construct. The CaMV35S promoter was then excised by *Sac*I/*Aat*II digestion and replaced with a PCR-amplified *MtARF5* promoter fragment using homologous recombination, yielding the final *ProMtARF5:CaARF5-GFP* plasmid. The construct was introduced into *Agrobacterium tumefaciens* strain EHA105 and transformed into the *Arabidopsis mp* mutant via the floral dip method. T0 transgenic seeds were sown in soil, and transformants were selected by spraying with Basta. In this experiment, the active ingredient of Basta was glufosinate ammonium, which was used for screening transgenic *Arabidopsis* at a working concentration of 20 mg/L.

## Data Availability

All relevant data are available within the manuscript and the Supplementary Materials.

## Supplementary Materials

The following supporting information can be downloaded at: https://www.mdpi.com/article/10.3390/plants15111708/s1, Figure S1: Sense probe control. In the in situ hybridization experiment, the sense probe was used as a negative control to assess signal specificity; Table S1: List of primer sequences used in this study; Table S2: *CaARF* family member ID and gene name; Table S3: Physicochemical properties of *CaARFs* gene family members in chickpea; Table S4: Conserved domains of *CaARF* family proteins in chickpea; Table S5: The location of *CaARF* in chickpea chromosomes; Table S6: Segmentally duplicated *ARF* gene pairs identified in the chickpea genome; Table S7: Orthologous relationships of *ARF* genes between chickpea and two representative plants.; Table S8: Detailed Information on the Analysis of Cis-acting Elements in Chickpea *ARF* Family Genes; Table S9: Statistics of cis-acting elements in chickpea *ARF* family genes; Table S10: Expression profiles of the *CaARF* family in different tissues of chickpea.
