# Supplementary material for: Genome-Wide Identification and Expression Analysis of the ARF Gene Family in Chickpea (Cicer arietinum)
Source: Plants (Basel). 2026 May 31;15(11):1708. doi: 10.3390/plants15111708 (PMC13258859; doi:10.3390/plants15111708)
Supplement: Supplementary file 1 [file plants-15-01708-s001.zip › plants-4324214-Figures S1.pdf]

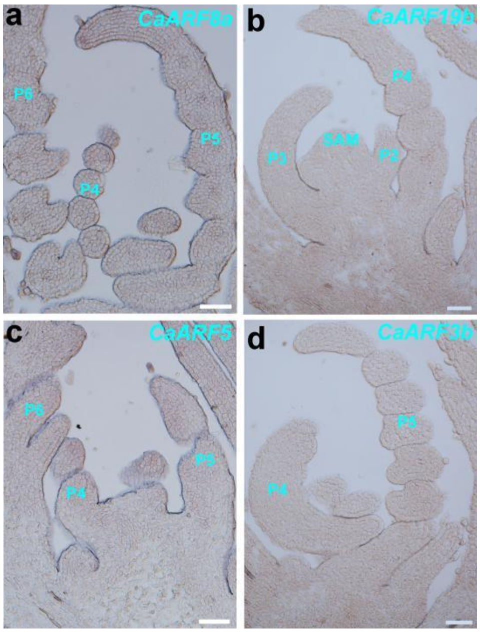

Figure S1: Sense probe control. In the *in situ* hybridization experiment, the sense probe was used as a negative control to assess signal specificity.
